# Metabolic Dysfunction-Associated Steatotic Liver Disease and Metabolic Dysfunction-Associated Steatohepatitis: The Patient and Physician Perspective

**DOI:** 10.3390/jcm12196216

**Published:** 2023-09-26

**Authors:** Wayne Eskridge, Donna R. Cryer, Jörn M. Schattenberg, Amalia Gastaldelli, Harmeet Malhi, Alina M. Allen, Mazen Noureddin, Arun J. Sanyal

**Affiliations:** 1 Fatty Liver Foundation, Boise, ID 83706, USA; 2 Global Liver Institute, Washington, DC 20016, USA; 3 Metabolic Liver Research Program, Department of Medicine, University Medical Center of the Johannes Gutenberg University, 155131 Mainz, Germany; 4 Cardiometabolic Risk Laboratory, Institute of Clinical Physiology, Italian National Research Council CNR, 00133 Pisa, Italy; 5 Division of Gastroenterology and Hepatology, Department of Medicine, Mayo Clinic, Rochester, MN 55901, USA; 6 Fatty Liver Program, Karsh Division of Gastroenterology and Hepatology, Cedar Sinai Medical Center, Los Angeles, CA 90048, USA; 7 Stravitz-Sanyal Institute of Liver Disease and Metabolic Health, VCU School of Medicine and Health System and Division of Gastroenterology, Department of Internal Medicine, VCU School of Medicine, Richmond, VA 23298, USA

**Keywords:** metabolic dysfunction-associated steatotic liver disease, metabolic dysfunction-associated steatohepatitis, fatty liver, liver disease, cirrhosis, primary care physicians, quality of life, outcome, advocacy

## Abstract

Diagnosing and managing metabolic dysfunction-associated steatotic liver disease (MASLD) remains a major challenge in primary care due to lack of agreement on diagnostic tools, difficulty in identifying symptoms and determining their cause, absence of approved pharmacological treatments, and limited awareness of the disease. However, prompt diagnosis and management are critical to preventing MASLD from progressing to more severe forms of liver disease. This highlights the need to raise awareness and improve understanding of MASLD among both patients and physicians. The patient perspective is invaluable to advancing our knowledge of this disease and how to manage it, as their perspectives have led to the growing recognition that patients experience subtle symptoms and that patient-reported outcomes should be incorporated into drug development. This review and expert opinion examine MASLD and metabolic dysfunction-associated steatohepatitis from the patient and physician perspective from pre-diagnosis to diagnosis and early care, through to progression to advanced liver damage. Specifically, the paper dives into the issues patients and physicians experience, and, in turn, what is required to improve diagnosis and management, including tips and tools to empower patients and physicians dealing with MASLD.

## 1. Introduction

Metabolic dysfunction-associated steatotic liver disease (MASLD, formerly non-alcoholic fatty liver disease, NAFLD) encompasses a spectrum of fatty liver diseases from steatotic liver disease (SLD), characterized by ≥5% hepatic steatosis, to metabolic dysfunction-associated steatohepatitis (MASH, formerly known as non-alcoholic steatohepatitis, NASH), with or without fibrosis, then cirrhosis, liver failure, and liver cancer at later stages [1]. MASLD and MASH represent a substantial burden to patients, society, and the economy, particularly in later disease stages characterized by advanced fibrosis [2], with the disability burden in these patients continuing to grow [3]. This highlights the need for timely diagnosis and management of the disease to reduce future healthcare and well-being costs [2]. Major challenges in diagnosing MASLD and MASH are limited awareness of the disease and its potential implications in patients [4], a lack of consensus on diagnostic tools [1,5], difficulty for patients and physicians in identifying symptoms and establishing their underlying cause [6], and lack of approved pharmacological treatments for MASLD and MASH [7]. As a result, MASLD is often diagnosed incidentally after results of blood tests or liver imaging come back as abnormal [8,9]. Raising awareness and improving understanding of MASLD remains crucial, as MASLD is under-diagnosed/recognized [10,11,12], and standardized and comprehensive models of care are lacking [13].

Gaining insight into the patient perspective of MASLD is critical to advancing our knowledge of how the disease presents, how it impacts patients, and, in turn, how it should be managed. This is demonstrated by the increasing evidence from qualitative studies and patient-reported outcomes that have shaped the growing recognition that MASLD is not asymptomatic [6,14,15]. Furthermore, with no approved pharmacological therapies, the patient perspective of MASLD can help inform clinical drug development and stakeholder decision making. Shedding light on the patient voice is therefore invaluable to bridging the gap between patients and physicians, ultimately improving patient care.

In this review and expert opinion, we explore the patient experience of MASLD and MASH from pre-diagnosis to diagnosis and early care, through to progression to advanced liver damage. An initial overview of the epidemiology of MASLD is provided to highlight the burden of the disease. Subsequently, we examine the challenges and issues patients and physicians experience and what is needed to improve diagnosis and management of the disease for both patients and physicians.

## 2. Epidemiology of MASLD

MASLD affects 25–30% of the global population [16,17], with prevalence ranging from 13.5% in Africa to 31.8% in the Middle East [16]. The prevalence of MASLD increases with age, and the mean and/or median age reported in studies ranges from 30.7–76.2 years [16]. MASLD is also more prevalent in individuals with cardiovascular/metabolic risk factors, including type 2 diabetes, obesity, hypertension, dyslipidemia, and metabolic syndrome [18,19]. The estimated global prevalence of MASLD in patients with type 2 diabetes is 55.5% [18]. The prevalence of MASLD is expected to rise alongside global increases in obesity and diabetes [20] and may be exacerbated in some individuals by weight gain related to home confinement during the COVID-19 pandemic [21].

Many patients with MASLD remain undiagnosed [22]. According to an analysis of primary care healthcare records from four large European databases, the estimated prevalence of MASLD (including MASH) was 1.85% in 2015 [10]. This is noticeably lower than the European prevalence estimate of 23.71% up to 2015 reported in a meta-analysis [16], showing how many patients are not officially diagnosed with MASLD/MASH. A separate study examining claims data from the US reported an estimated MASLD/MASH prevalence of 5.7% in 2015 [23], lower than the North American prevalence estimate of 24.13% up to 2015 in the abovementioned meta-analysis [16] and US prevalence estimate of 25.3% in an analysis of the National Health and Nutrition Examination Survey database from 2017–2018 [24]. In a population-based screening study in the US in high-risk individuals, as determined by FibroScan^®^ scores, 40% of participants met the study criteria for MASLD with a steatosis grade of S3, while 5% of the population had a fibrosis score of F3 or F4 and therefore met the study criteria for MASH [25]. These findings demonstrate that patients with early- and late-stage disease are under-diagnosed [25]. Consistent with this, a population-based study in Germany using surrogate scores of hepatic steatosis (fatty liver index) and fibrosis (Fibrosis-4 index) estimated that hepatic steatosis was prevalent in 37.5% of the population and advanced fibrosis in 1.1% [26].

A retrospective analysis of observational clinical trials estimated that one-third of patients with MASLD develop progressive fibrosis, just under half remained stable, and fibrosis improved in one-fifth of patients [27]. In a prospective cohort study in patients with MASLD and MASH, fibrosis was reported to progress by ≥1 stage in 33.9% and regress by ≥1 stage in 30.0% [28]. A prospective multicenter study estimated an increased incidence rate of liver-related complications per 100 person-years with fibrosis stage (F0 to F2 vs. F3 vs. F4) for variceal hemorrhage (0.00 vs. 0.06 vs. 0.70), ascites (0.04 vs. 0.52 vs. 1.20), encephalopathy (0.02 vs. 0.75 vs. 2.39), and hepatocellular cancer (0.04 vs. 0.34 vs. 0.14) [29]. In the US, MASH is the leading cause of liver transplants in women and second leading cause in men [30].

Patients with MASLD are more likely to die from any cause and liver-related events compared with those without MASLD, with an estimated incidence rate ratio of 1.05 and 1.94, respectively [16]. A prospective multicenter study estimated the incidence rate of deaths from any cause at 0.32 deaths per 100 person-years for stage F0–F2, rising to 0.89 deaths per 100 persons-years for stage F3 and 1.76 deaths per 100 person-years for stage F4 in patients with MASLD [29]. A population-based cohort study reported mortality risk increased with MASLD severity, with the absolute excess risk of death from any cause after 20 years estimated at 10.7% for patients with simple steatosis, 18.5% for MASH without fibrosis, 25.6% for fibrosis without cirrhosis, and 49.4% for cirrhosis when compared to controls [31]. Collectively, these data show how MASLD can progress to serious complications, which in turn increase patients’ risk of death.

## 3. The Patient Journey: Pre-Diagnosis

### 3.1. Patient Experience

Globally, there remains a lack of public awareness of MASLD and how common it is [32,33,34].

“*When people say, ‘I have XYZ cancer,’ this has meaning. When people say, ‘I have ‘NAFLD’ or ‘I have ‘NASH,’ people have no clue what this means*”—80-year-old male with MASH and stable, compensated cirrhosis, with no comorbidities.

**Figure 1:**
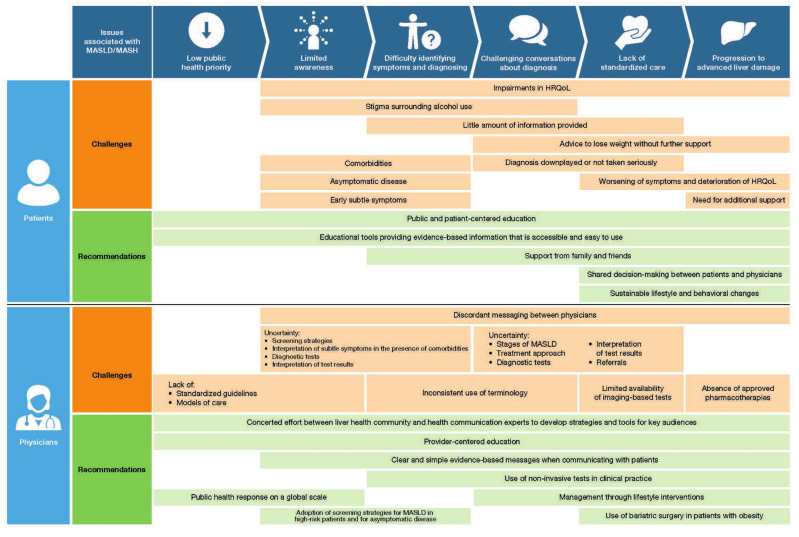
Issues associated with MASLD/MASH and corresponding challenges and recommendations for patients and physicians [1,4,5,6,7,11,12,14,34,39,40,41,42,43,44]. HRQoL, health-related quality of life; MASLD, metabolic dysfunction-associated steatotic liver disease; MASH, metabolic dysfunction-associated steatohepatitis.

Patients at high risk of MASLD, such as those with diabetes and obesity, remain largely unaware of the disease and/or that their conditions are risk factors for MASLD [35,36,37]. This is likely due to a variety of factors, including limited screening for the disease and difficulty in identifying early subtle symptoms (Figure 1) [1,4,5,6,7]. It is important for healthcare professionals to understand that a lack of awareness corresponds with a limited level of knowledge of MASLD among the general population. We therefore suggest that when communicating with patients, clear and simple messages are critical to help improve their understanding of MASLD.

“*The combination of my emotional state and his use of technical terms unknown to me resulted in a rather complete lack of communication between us even though we had both tried. Getting the timing and balancing the emotional state of the patient are crucial to the teachable moment and needs specific awareness of what the patient understands of the language used*”—80-year-old male with MASH and stable, compensated cirrhosis, with no comorbidities.

On a broader scale, raising awareness of the disease as a public health issue is crucial (Figure 1). Public and patient-centered education on (i) what MASLD is and its consequences, (ii) who is at an increased risk of developing MASLD, and (iii) what can be done to prevent and/or treat the disease is required to improve public awareness [34]. Educational tools should provide patients with evidence-based information free of commercial bias and be accessible and easy to use (Figure 1); one example of this is a recent patient guideline that was developed for patients at risk of and/or with MASLD [38]. As outlined in the consensus recommendations for an MASLD public health agenda, a concerted effort between the liver health community and health communication experts will be key to developing effective and practical strategies and tools to engage key audiences [34].

**Figure 2:**
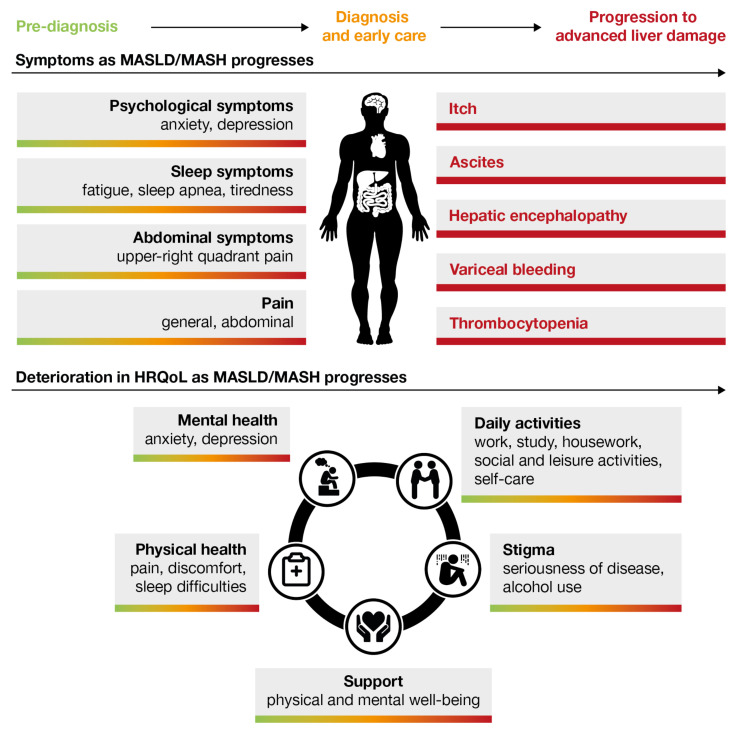
Overview of symptoms and HRQoL as MASLD/MASH progresses [57,59,60,61,62,63,64,65,66,67]. HRQoL, health-related quality of life; MASLD, metabolic dysfunction-associated steatotic liver disease; MASH, metabolic dysfunction-associated steatohepatitis.

MASLD is often described as being asymptomatic in most patients [45,46]. However, emerging evidence suggests that some patients with MASLD experience early subtle symptoms and reduced health-related quality of life (HRQoL; Figure 1 and Figure 2) [47,48,49,50,51]. Sleep, pain, psychological, and abdominal symptoms are among the most commonly reported symptoms in patients with MASLD [52,53]. Fatigue, which may be related to sarcopenia in some patients [47,48], is another symptom commonly reported, even in the early stages of MASLD, and negatively impacts HRQoL [49,50,51]. In a study in Cuba, more than a third of patients with MASLD reported experiencing anxiety, depression, abdominal pain, sleep apnea, and fatigue, although the underlying cause of symptoms was not established [54]. Some patients with MASLD report experiencing abdominal pain, some specifically in the upper-right quadrant of their abdomen [15,39,55]. This pain can be attributed to distension of the capsule that surrounds the liver, known as the Glisson’s capsule [56]. This is a thin tissue that contains pain receptors, which can sense swelling or distension of the liver [56]. It is the authors’ opinion that although this pathological mechanism has been proposed as the cause of abdominal pain in patients with MASLD, for most symptoms and patient-reported outcomes establishing a causal link with the pathology of MASLD remains a challenge. The main reason for this is that these subtle symptoms are often attributed to or associated with other more identifiable conditions, such as obesity, diabetes, and irritable bowel syndrome [6,14,16,57,58]. This makes it difficult for patients to recognize subtle symptoms that may indicate they have MASLD and distinguish these symptoms from other conditions they may have, as well as it potentially being difficult for healthcare professionals to identify the cause of these symptoms in patients with multiple comorbidities [6,14] (Figure 1).

On the other hand, some patients with MASLD do not experience any symptoms in the earlier stages of the disease [39]. In the experience of the authors, patients who are symptom-free or otherwise in good health may be hesitant to accept their MASLD diagnosis (Figure 1). However, even asymptomatic patients can experience more severe forms of MASLD and be at risk of complications [25]. Communicating with patients about risk factors for liver disease as well as the risks that can arise from liver disease is therefore essential.

Besides early subtle symptoms, patients with MASLD experience more impairments in HRQoL compared with the general population [59,60]. The extent of impairment may be related to the severity of MASLD, as higher levels of liver fat content are associated with greater impairments in HRQoL [57]. MASLD impacts various aspects of HRQoL, with patients reporting negative impacts on their physical and mental health as well as activities of daily living [59,60,61,62]. Another factor affecting HRQoL is the stigma surrounding liver disease, with family and friends but also physicians assuming patients are likely alcoholic and questioning patients’ honesty regarding their alcohol intake [63]. Collectively, these findings demonstrate the burden of MASLD on patients’ physical and mental well-being, which can occur even in earlier stages of the disease, prior to diagnosis (Figure 2).

### 3.2. Physician Experience

The challenges in disease recognition and diagnosis and lack of approved pharmacological therapies as outlined above, combined with competing medical priorities, may explain why the prevalence of MASLD is underestimated in primary care [4,12]. Limited awareness of MASLD and how to manage the disease is partially because MASLD is a low public health priority [40,41,42] (Figure 1). A study that surveyed MASLD experts from 29 European countries concluded that a public health response to MASLD in Europe is lacking, with regard to national policies and registries, clinical guidelines, awareness campaigns, and civil society involvement [41]. A subsequent global study reported that none of the 102 countries surveyed had a national strategy for MASLD in 2020 [42]. As a result, there are limited national guidelines and models of care for MASLD [13,42] and no unified approach to identifying and managing these patients [1,68,69,70]. For example, guidelines from the American Diabetes Association, European Association for the Study of the Liver 2016, and Asia-Pacific Working Party recommend that physicians should screen patients at high risk for MASLD, specifically those with cardiovascular and/or metabolic risk factors such as obesity and diabetes [68,69,70]. In contrast, the American Association for the Study of Liver Diseases and the UK National Institute for Health and Care Excellence do not formally recommend screening but rather state that physicians should be aware that these patients are at higher risk of MASLD [1,71].

A lack of consensus is echoed in findings from questionnaires and interviews with primary care physicians as well as analyses of electronic patient records, which suggest physicians are uncertain about how to interpret abnormal liver tests, what non-invasive tests are available and how to use them, which are the most appropriate to detect clinically significant findings, and when to refer patients to specialists [4,11,12,40,43,44] (Figure 1). These uncertainties translate to MASLD not being recognized or evaluated properly in primary care. For example, one study examining medical records from a primary care setting in the US found that only the extent of abnormally elevated liver enzymes was associated with patients receiving MASLD care [11]. However, patients with normal and abnormal alanine aminotransferase levels can have a similar extent of liver damage [39]. While guidelines promote confidence for some physicians, others report feeling a lack of confidence in interpreting abnormal liver function test results and deciding how to proceed due to time constraints, limited knowledge of additional tests, and uncertainty regarding referrals to specialists [40]. We suggest that physicians with limited knowledge may struggle to communicate about MASLD with patients. A public health response on a global scale is critical to fueling awareness and understanding of MASLD for both patients and providers [34].

## 4. The Patient Journey: Diagnosis and Early Care

### 4.1. Patient Experience

Diagnosis and early care occur in various care settings and by different means. Most patients with MASLD/MASH are diagnosed and managed in primary care, with a smaller portion by specialists, including hepatologists, gastroenterologists, endocrinologists, and cardiologists [52,72]. In turn, various tools are used to diagnose MASLD/MASH including blood tests, imaging, and biopsy [14,16,52]. As a result, conversations about MASLD/MASH between patients and physicians vary (Figure 1) [14,73]. Some patients report that they received little information from their physician after receiving a “fatty liver” diagnosis, including on the severity of the disease and treatment/management options [14,73]. Other patients report that physicians advised that MASLD was nothing to be concerned about relative to other more important health conditions, and some report feeling that their physicians did not take their diagnosis seriously or downplayed it [14,73]. Patients also report being advised to lose weight but not receiving further support; this was perceived by some as the physician downplaying their disease and considering it to be the same as being overweight [14,73].

The diagnosis of MASLD/MASH is often a long process over several years. One possible reason for this is that some patients are asymptomatic while others experience subtle symptoms [14,39]. We also suggest that the diagnosis of MASLD/MASH is further complicated by physicians describing the diseases inconsistently; some use the terms “fatty liver”, “fatty liver disease”, “NAFLD”, and “NASH” interchangeably (Figure 1). Although MASLD can be used as an umbrella term for the spectrum of liver diseases from SLD to cirrhosis, each subtype of MASLD is histologically distinct; using the correct terminology is therefore important, particularly when the disease advances to more severe forms [1]. For example, some physicians tell patients that they have a fatty liver and that this is nothing to worry about, so patients do little about it. In contrast, if physicians tell patients that they have a disease, they may be more proactive in making lifestyle changes that could delay disease progression [74,75,76].

“*Doctor told me 10 years ago I had a little fatty liver, but I never realized it would progress*”—80-year-old male with MASH and stable, compensated cirrhosis, with no comorbidities.

Further confusion surrounding the terminology may result from the recent changes from “NAFLD” and “NASH” to “MAFLD” and “MASH” [77,78,79]. Since terminology can influence patients’ perceptions and behaviors toward a disease, we would encourage physicians to use precise terms when communicating with patients about the stage of their disease.

In addition to the early stages of MASLD, patients experience impaired HRQoL throughout the disease process [57,64,67] (Figure 2). Impacts on physical and/or mental health include pain, discomfort, sleep difficulties, anxiety, and depression [52,53]. MASLD and MASH can also negatively impact patients’ daily activities, such as work, studying, and housework, and social and leisure activities, including self-care [14,52,53]. As a result, some patients report needing or feeling the need for additional support to manage their disease and well-being [14]. Perceived social support can in turn influence patients’ HRQoL [80] and behavioral therapy and counseling programs can help patients with lifestyle changes [81,82]. Regarding disease severity, patients with MASH experience greater impairment in HRQoL than patients with MASLD without MASH [60]. It remains unclear whether patients with more advanced fibrosis experience greater impairments in HRQoL than those in the earlier stages of MASLD [83]. Nonetheless, emerging evidence shows patient-reported outcomes, such as HRQoL, are associated with histological and clinical features of MASLD and MASH [57,84].

We suggest that the reactions of friends and family may be another component that impacts patients’ HRQoL after diagnosis of MASLD. In our experience, some patients describe this as being the hardest part of the disease, as family and friends do not believe the seriousness of the disease since patients may appear physically well. Stigma around alcohol use can also impact patients’ HRQoL, as friends, family, but also physicians may make assumptions and judgments of patients’ alcohol intake. This highlights how physical components of the disease and societal factors contribute to reduced HRQoL in patients diagnosed with MASLD and MASH (Figure 2).

### 4.2. Physician Experience

In the opinion of the authors, discordant messaging within primary care and between primary care physicians and specialists appears to be an ongoing issue when it comes to managing patients with MASLD and MASH (Figure 1). This suggests that patients might frequently encounter conflicting messages and advice from different physicians. One reason for this is limited awareness among some physicians about the differences between MASLD and MASH [85]. Regarding management of patients, in a study in the UK, physicians interviewed expressed that they were unaware of structured approaches for following-up patients and voiced the need for systems in place to provide more standardized care [40]. A nationwide survey in the US found that across various specialties (including general internal medicine, pediatric medicine, family medicine, geriatrics, endocrinologists, and cardiologists), physicians reported the most common barrier to a diagnosis was uncertainty regarding the optimal treatment approach and they expressed differing opinions on screening for MASLD and dietary recommendations [86]. Another challenge for physicians is managing and supporting patients’ lifestyle changes due to the struggles patients themselves face with adhering to lifestyle interventions because of time constraints or lack of interest, as well as a lack of support from external services [86].

Awareness of liver disease has improved in recent decades as more diagnostic tools have become available. Recognition of MASLD as a disease emerged in the 1980s [87]. Non-invasive tests were developed in the early 2000s [88,89] and are now recommended for screening for fibrosis in clinical practice due to their low cost and wide availability, and not all patients with MASLD require a liver biopsy [1,5,90]. However, liver biopsy remains the only definitive tool for diagnosis of MASH, making this a barrier to diagnosing this specific stage of the liver disease [1,5,90].

Where previously there were limited data available on the effectiveness of lifestyle interventions [91], now lifestyle interventions have a strong evidence base and are recommended in several guidelines [1,68,90]. Despite there being no approved pharmacological treatments for MASLD and MASH [7], lifestyle interventions can prevent and reverse disease progression. In patients with MASLD and MASH, both diet and exercise in the absence or presence of weight loss can reduce levels of liver fat and liver enzymes, which are markers of inflammation when elevated [74,75,76,92,93]. Evidence also suggests that fibrosis regresses when individuals lose 10% of their body weight [94,95,96,97]. Furthermore, in patients with risk factors for MASLD, such as type 2 diabetes and obesity, lifestyle interventions have been shown to reduce liver fat content and the risk of developing MASLD [98,99,100]. Patients with MASLD and risk factors, including type 2 diabetes, obesity, hypertension, and/or dyslipidemia, are also at a risk of progressing to more severe complications, including MASH and fibrosis, and death [1,19,27,101,102,103,104]. Therefore, we suggest that identifying individuals at risk of MASLD or patients with MASLD as early as possible is crucial to enact preventive measures and empower patients to make lifestyle changes to prevent disease development and progression. Slowing the progression of the disease to more advanced stages is also critical since fibrosis is the main determinant of liver-related morbidity and mortality in MASLD [105,106,107]. Bariatric surgery has also emerged in the last decade as a tool in the management of MASH for improving histopathological features and markers of MASLD, but randomized controlled trials are lacking [108,109,110]. Nonetheless, guidelines recommend that it should be considered in patients with obesity [1,70].

## 5. The Patient Journey: Progression to Advanced Liver Damage (Bridging Fibrosis and Cirrhosis)

### 5.1. Patient Experience

As MASLD and MASH progress to bridging fibrosis and cirrhosis, the severity of patients’ symptoms and impacts on HRQoL tend to worsen (Figure 2). In a study examining patients who received a liver transplant for cirrhosis arising from MASLD, 68.5% were unaware of their pre-existing MASLD until presenting with new-onset ascites, hepatic encephalopathy, variceal bleeding, and/or thrombocytopenia [66]. One study found significant fatigue and itch occurred in approximately one-third of patients with bridging fibrosis/cirrhosis; patients with cirrhosis experienced more fatigue than those with bridging fibrosis but the difference did not reach a clinically important threshold [6]. Patients who experienced significant fatigue or itch reported significant impairments in HRQoL, with the greatest impairment reported in those who experienced both symptoms [6]. Another study of patients with bridging fibrosis and/or compensated cirrhosis found that HRQoL was reduced in comparison with the general population [111]. Similarly, patients with cirrhosis showed greater impairments in HRQoL versus patients with bridging fibrosis in this study [111]. In a targeted literature review examining 31 studies of patients with MASH-related cirrhosis, HRQoL was lower than in patients with non-cirrhotic MASH and the general population [65]. Key symptoms reported among the studies, in patients with cirrhosis, were abdominal symptoms, abdominal pain, lack of energy, tiredness, pain, and sleep symptoms [65]. Four studies reported gastro-esophageal varices in patients with cirrhosis [65]. A separate study reported that patients with cirrhosis with MASH or hepatitis C were more likely to experience general pain and abdominal pain than those with alcohol-related liver disease [112]. Collectively, these studies highlight how symptoms worsen and HRQoL deteriorates as MASLD and MASH progress to more advanced liver damage.

### 5.2. Physician Experience

In primary care, one of the barriers to assessing bridging fibrosis and cirrhosis associated with MASLD and MASH is an unfamiliarity with tests available for evaluating fibrosis, how to interpret the test results, and when to refer patients to specialists [11,12,40,44]. Analyses of medical records and findings from surveys suggest some physicians are uncertain about the Fibrosis-4 score and NAFLD fibrosis score [11,12,43,44], despite several guidelines recommending these tests as first-line non-invasive scores for assessing fibrosis [1,5,90]. Interviewed primary care physicians from the UK voiced that investigations beyond a standard liver function test were potentially more suited for specialists to conduct due to time pressures, lack of specialist knowledge, and limited access to imaging-based tests [40]. This highlights another issue, namely the limited availability of imaging-based tests, including vibration-controlled elastography and magnetic resonance imaging methods, in primary care settings [1,68,71,113]. Finally, we suggest that in the absence of available pharmacotherapies and with the challenges associated with prescribing and supporting lifestyle changes, some physicians may feel less motivated to address MASLD and/or MASH (Figure 1).

## 6. Recommendations for Managing MASLD: Practical Tools and Tips

### 6.1. Patient Perspective: Commentary on Patient Journey and How to Improve the Patient/Physician Relationship

In the experience of the authors, it is easy to dismiss low-grade subtle symptoms, such as fatigue, pain, sleep disturbances, and psychological and abdominal symptoms, as signs of aging or lifestyle choices. Recognizing symptoms that impact our daily lives or general well-being is important, as they may indicate an underlying health condition, such as MASLD. Equally important is that individuals with cardiovascular and metabolic comorbidities, namely diabetes, obesity, hypertension, dyslipidemia, and metabolic syndrome, are made aware of their increased risk of MASLD. Furthermore, seemingly healthy individuals can also develop MASLD without experiencing any symptoms. Therefore, it is critical for patients to understand that MASLD can progress to serious complications and death, despite initially not showing any severe symptoms or symptoms at all [25].

Support from family, friends, and/or caregivers is another critical aspect to improving the HRQoL of patients with MASLD/MASH. Helping individuals manage their disease, understanding the stigma surrounding MASLD/MASH, encouraging and adapting to their lifestyle changes, and supporting their mental health and well-being can all make a difference in how patients cope with their diagnosis (Figure 1).

Raising awareness and educating patients about the progressive nature of MASLD is crucial, as lifestyle modifications can reduce the risk of MASLD developing and prevent MASLD from progressing to more serious complications. General advice to lose weight is not an effective strategy. Rather, focus should be on encouraging patients to make long-term lifestyle and behavioral changes, by initially identifying barriers to weight loss [114,115]. Diet and exercise recommendations should therefore be tailored to individuals’ situations and priorities to ensure lifestyle changes are sustainable [114,115]. This means engaging patients in the decision-making process [114,115,116]. According to the Guideline for the Management of Overweight and Obesity in Adults, a comprehensive lifestyle intervention involving trained interventionists or nutrition professionals is recommended for weight loss [114]. This should comprise a diet plan, physical activity program, and behavioral strategies. Programs more than 1 year in length with contact at least monthly are key to weight-loss maintenance, with successful weight maintenance requiring continued support from a multidisciplinary team, including medical, nutrition, and behavioral experts, to navigate patients’ weight changes and adapt their approaches accordingly [114]. Consistent messaging from physicians regarding lifestyle modifications would also be valuable.

### 6.2. Physician Perspective: Commentary on Diagnosing MASLD and MASH

In the opinion of the authors, there is a need for greater understanding of MASLD and MASH and screening in high-risk populations and for asymptomatic disease (Figure 1). MASLD and MASH are prevalent in individuals with cardiovascular and/or metabolic conditions, including in those who are asymptomatic [18,25]. Individuals with cardiovascular and/or metabolic risk factors also appear to be at an increased risk of developing more severe forms of MASLD [19]. Screening for MASLD in at-risk populations is therefore important.

Although there are no approved pharmacological therapies for MASLD and MASH [7], lifestyle modifications can significantly improve liver and metabolic outcomes and are therefore a critical component to managing patients with MASLD [74,75]. However, these can be challenging for physicians to advocate, with physicians stating patient time constraints, limited patient interest, and lack of access to dietitians and lifestyle programs hindering their referrals to these support services [86].

Liver biopsy is an invasive procedure associated with risks [1,68,71]; patients express concerns and are hesitant to receive a biopsy [14]. However, there are recent efforts to improve diagnosis and risk stratification, including new tools available to physicians. Non-invasive tests are recommended for screening for fibrosis [1,5,90] and represent valuable diagnostic tools in primary and secondary care settings [34]. Nonetheless, more accurate non-invasive tests are still required [34], with two large consortium projects currently underway in the US and EU investigating biomarkers in MASLD [117,118].

### 6.3. Tools to Empower Patients and Physicians

#### 6.3.1. Patients

The authors encourage patients to ask their primary care physician the following questions during a check-up or upon a first diagnosis of MASLD.

- I have diabetes/obesity/high cholesterol/hypertension—should I be checked for MASLD?
- What tests can you do to figure out if I have MASLD?
- Have you checked if something else could be causing my liver damage?
- Have you checked if I have any scarring in my liver?
- What are the risks of MASLD?
- What symptoms or signs should I look out for that MASLD is getting worse?
- Can MASLD be reversed?
- Will MASLD shorten my life?
- How does MASLD affect other health problems I have?
- Are any of the other medications I take a problem?
- Where can I find more information on managing or preventing MASLD/MASH?
- What lifestyle changes do I need to make?
- What services are available to help me make lifestyle changes?
- What treatments or tools are available to me besides lifestyle changes?
- Are there any other doctors I should reach out to about my MASLD?

#### 6.3.2. Physicians

Below are the authors’ key recommendations for physicians managing patients with MASLD.

- MASLD is associated with an increased risk of type 2 diabetes, cardiovascular disease, dyslipidemia, and sleep disturbances. All associated comorbidities should be controlled to effectively manage MASLD.
- Symptoms can be difficult to recognize. There are a variety of non-invasive tests available to assess the amount of liver fat and fibrosis, which are safer and can provide a better overall picture compared with a liver biopsy.
- It is important to assess the stage of liver fibrosis and its progression over time to determine an individual’s risk for developing cirrhosis and other related complications.
- Lifestyle changes are currently the keystone strategy for managing MASLD. In patients with obesity-associated MASLD, reducing body weight by 5–10% is associated with improvement of MASLD. Sustained lifestyle changes can prevent MASLD from progressing and reverse MASLD in the early stages of the disease.
- Patients are encouraged to develop methods to control risk factors and implement sustainable lifestyle changes. However, comprehensive, structured lifestyle interventions comprising a dietary, physical, and behavioral plan that offer continued support from a multidisciplinary team are critical to helping patients with weight loss and maintenance.

## 7. Conclusions

Experiences from patients and physicians alike show that MASLD and MASH are progressive diseases with symptoms that adversely affect HRQoL. A global public health response to MASLD, involving patient- and provider-centered education, needs to be made a priority to improve awareness of the disease and how to diagnose and manage it, issues highlighted by both patient and physician perspectives. This will help bridge the gap between patients and physicians as well as among healthcare professionals, where a unified approach is missing yet required to help diagnose patients in a timely manner and monitor their disease over time. As sustainable lifestyle changes are critical to delay and prevent disease progression, comprehensive support from a multidisciplinary healthcare team as well as friends and family is key to helping patients achieve weight loss and maintenance in the long term. Clear understanding and consideration of the patient perspective in MASLD are imperative. Despite the many challenges and problems that remain to be addressed, the key questions for patients and key recommendations for physicians provided in this review contribute to the tools available to individuals dealing with MASLD.

## Data Availability

Not applicable.

## Abbreviations

HRQoL, health-related quality of life; MAFLD, metabolic-associated fatty liver disease; MASH, metabolic dysfunction-associated steatohepatitis; NAFLD, non-alcoholic fatty liver disease; NASH, non-alcoholic steatohepatitis; SLD, steatotic liver disease.
